# The underappreciated, underrecognized problem of fourth chromosome trisomy in *Drosophila melanogaster* stocks and a simple, general method for building diplo-4 stocks from triplo-4 stocks

**DOI:** 10.17912/micropub.biology.001606

**Published:** 2025-04-29

**Authors:** Kevin R. Cook

**Affiliations:** 1 Bloomington Drosophila Stock Center, Department of Biology, Indiana University, Bloomington, Indiana, United States

## Abstract

Trisomy of the small, fourth chromosome is common in *Drosophila melanogaster* stocks. Even though the presence of an extra chromosome can confound the interpretation of experimental crosses, many Drosophila geneticists are unaware of this potential problem. Here I describe a simple method employing a mutation in a haploinsufficient gene to recognize fourth chromosome trisomy and establish disomic stocks from trisomic stocks.

**Figure 1. Using a Minute mutation to establish stocks disomic for the fourth chromosome from trisomic stocks:**
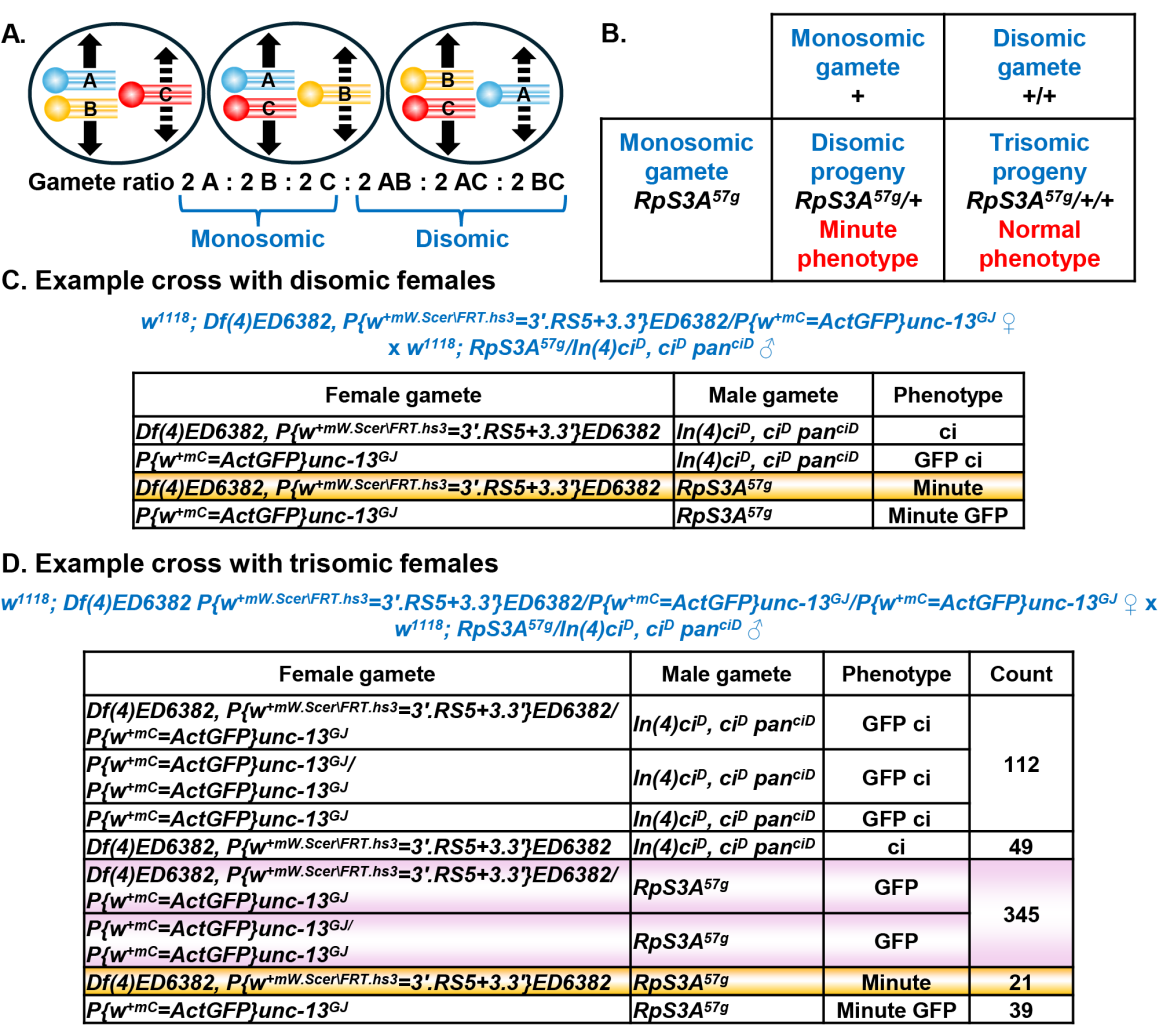
A. In meiosis I, the three chromosomes in a trisomic fly (shown here as A, B and C in different colors) can form a paired bivalent and an unpaired univalent in three ways. The two univalents that pair will segregate from each other (solid arrows). The unpaired univalent can segregate with either of the other univalents (dashed arrows). Six segregation patterns are possible to produce six kinds of gametes (A, B, C, AB, AC and BC). (Each kind of gamete can be formed from two of the six segregation patterns.) If there are no biases in chromosome behavior, the six kinds of gametes are produced in equal frequencies at shown by this gamete ratio—with half the gametes acquiring a single chromosome (monosomic) and half acquiring two chromosomes (disomic). B. As shown in this table, a gamete carrying the haploinsufficient Minute mutation *RpS3A ^57g^* produces a disomic, Minute progeny when it combines with a monosomic gamete, but produces a trisomic, non-Minute progeny when it combines with a disomic gamete. C. This example cross shows the progeny produced when a disomic fly mates with a *RpS3A ^57g^* fly. All progeny inheriting *RpS3A ^57g^* show Minute phenotypes. It is easy to identify disomic flies inheriting the chromosome of interest (in this case, flies with the deletion chromosome, highlighted in yellow). D. This example cross shows the progeny produced when a trisomic fly mates with a *RpS3A ^57g^* fly. Trisomic progeny inheriting two fourth chromosomes from the trisomic parent and a chromosome bearing *RpS3A ^57g^* will not be Minute (highlighted in pink), whereas disomic progeny inheriting a single fourth chromosome and a chromosome bearing *RpS3A ^57g^* will be Minute (highlighted in yellow). The presence of trisomic, non-Minute progeny inheriting *RpS3A ^57g^* shows unequivocally that the parent was trisomic. Even though the disomic, Minute progeny typically constitute the smallest classes, those with the desired chromosome (in this case, flies with the deletion chromosome, highlighted in yellow) may be crossed to balancer-bearing flies to recover male and female sibling progeny to use in establishing a new disomic stock.

## Description

While diploid flies with an extra second or third chromosome invariably die early in development and those with an extra *X* chromosome have low viability, trisomic flies with an extra fourth chromosome survive at rates equivalent to normal, disomic flies and show nearly imperceptible phenotypic differences (Morgan et al. 1925). Flies trisomic for the fourth chromosome arise from nondisjunction, which occurs spontaneously in roughly 1 out of every 500 meioses in normal flies (Baker and Carpenter 1972)—meaning that trisomic flies appear frequently in stocks. Once a trisomic fly appears, it can produce trisomic progeny. ( Figure 1A shows that half the gametes of a trisomic fly are disomic if meiotic chromosome pairing and segregation are random.) If trisomy provides a selective advantage, trisomic flies can become a large proportion of the stock population. For example, it is likely that an extra normal chromosome can ameliorate deleterious effects associated with a deletion chromosome being heterozygous to a single normal chromosome. Indeed, in my recent work with the Fourth Chromosome Resource Project (Weasner et al. 2025), I found that several deletion stocks in the Bloomington and Kyoto stock centers were trisomic.

It is easy to see how using a stock with an unexpected, extra fourth chromosome can lead to confusing experimental results. Extra gene copies can cause higher-than-usual gene expression—with downstream consequences to gene regulation across the genome including modified phenotypes. Since most fourth chromosomes do not have dominant phenotypic markers, extra fourth chromosomes can be inadvertently carried along in crossing schemes and end up contaminating newly built stocks or making mutations appear to complement when they really do not.

Trisomy can be recognized from altered chromosome inheritance: each distinct fourth chromosome does not appear in half of the gametes as expected in disomic flies. Simplistically, one would expect that two of the three fourth chromosomes in a trisomic individual would pair at random and segregate from each other and that the segregation of these two chromosomes would not influence the segregation of the remaining chromosome ( Figure 1A ). In fact, such random, unbiased chromosome behavior, producing six gamete types in equal numbers, is rarely, if ever, seen. Sturtevant (1936) showed that, in general, two chromosomes preferentially pair and the remaining chromosome tends to segregate with one of them—leading to unequal numbers of the six gamete types. For example, I saw, in several dozen crosses with a trisomic *Df(4)ED6382/P{ActGFP}unc-13 ^GJ^ /P{ActGFP}unc-13 ^GJ^* stock, that progeny from monosomic *Df(4)ED6382* gametes were generally underrepresented.

To establish a disomic stock from a trisomic stock, one simply needs to identify a disomic fly with the chromosome of interest and mate it to disomic, balancer-bearing flies to recover siblings that can be mated to produce a stable stock. For example, I recovered the deletion chromosome from a trisomic *Df(4)ED6380/l(4)102EFf ^1^ /l(4)102EFf ^1^* stock (which the DrosDel Project (Ryder et al. 2007) established using the unmarked lethal mutation *l(4)102EFf ^1^* ) by crossing trisomic females to males homozygous for the recessive *ey ^1^* mutation. Disomic *Df(4)ED6380/ey ^1^* male progeny were easy to identify from their *eyeless* phenotypes and then to mate to *In(4)ci ^D^ , ci ^D^ pan ^ciD^* females to recover *Df(4)ED6380/In(4)ci ^D^ , ci ^D^ pan ^ci^* sibs for mating. Since noncomplementation is often not a realistic option for identifying disomic progeny carrying a chromosome of interest, fly geneticists have typically crossed flies from a trisomic stock to balancer-bearing flies and blindly set up multiple crosses with individual progeny in the hope of establishing at least one line with a disomic founder. For example, I attempted to establish disomic, deletion stocks by outcrossing *Df(4)ED6369/l(4)102EFf ^1^ /l(4)102EFf ^1^* and *Df(4)ED6384/l(4)102EFf ^1^ /l(4)102EFf ^1^* flies to disomic, balancer-bearing flies, backcrossing individual male progeny to balancer-bearing females and establishing sib matings. While this brute-force approach can undoubtedly be successful with enough effort, I found, after establishing roughly a dozen lines per deletion, that the *l(4)102EFf ^1^* chromosome had been present in every founder male and that, consequently, every line was trisomic. Preferential segregation of the deletion chromosomes with the *l(4)102EFf ^1^* chromosome in the original trisomic stocks (à la Sturtevant) likely reduced my chances of success.

After my frustrating experiences with those two trisomic stocks, I wanted a simpler solution to establishing disomic stocks from trisomic stocks, so I devised a general approach that employs a mutation in a haploinsufficient gene on the fourth chromosome, *RpS3A* (Morgan et al. 1926). *RpS3A* encodes a protein component of ribosomes and, when only a single functional gene copy is present, flies display Minute phenotypes (short and thin bristles, small body, reduced fertility and reduced viability) (Marygold et al. 2007). As shown in Figure 1B, the *RpS3A ^57g^* loss-of-function mutation (Hochman et al. 1964) gives Minute phenotypes when it is combined with a single fourth chromosome but gives no mutant phenotypes when it is combined with two other fourth chromosomes. From comparing the example cross of disomic flies to *RpS3A ^57g^* flies in Figure 1C to the example cross of trisomic flies to *RpS3A ^57g^* flies in Figure 1D, it is evident that parents from a trisomic stock will produce a class of non-Minute progeny (highlighted in pink in Figure 1D ) that is not present among the progeny of disomic parents. Furthermore, it is easy to select the appropriate class of disomic, Minute progeny (highlighted in yellow in both Figures 1C and D) to found a disomic stock. From the progeny of the cross shown in Figure 1D, I crossed the *w ^1118^ /Y; Df(4)ED6382/ RpS3A ^57g^* males to y ^1^ w ^*^ ; TI{GMR-HMS04515}Gat ^eya^ /In(4)ci ^D^ , ci ^D^ pan ^ciD^ females (Nyberg et al. 2020) to establish a disomic w ^*^ ; *Df(4)ED6382/In(4)ci ^D^ , ci ^D^ pan ^ciD^* stock via sib mating. I have also used this approach to establish disomic stocks for *Df(4)ED6369* and *Df(4)ED6384* .

The *RpS3A ^57g^* mutation was caused by a *Doc* transposable element insertion upstream of *RpS3A .* It drastically reduces *RpS3A* mRNA expression (van Beest et al. 1998; Kronhamn and Rasmuson-Lestander 1999). Even though *RpS3A ^57g^* is likely a null allele, the *RpS3A ^57g^* stock grows better than most stocks for multigene deletions removing *RpS3A* . *RpS3A ^57g^* heterozygotes had lower viability than other progeny classes in Figure 1D, but I had little trouble using heterozygous males to establish new disomic stocks. The Minute bristle phenotypes are much easier to score than the other Minute phenotypes, and the bristle phenotypes are easier to score in females than males. I generally cross a single Minute male to multiple females to found a line and set up multiple lines in case I mistake non-Minute males for Minute males. A deletion for *RpS3A* could probably be substituted for *RpS3A ^57g^* in isolating disomic progeny, but I would anticipate somewhat lower viability with deletion heterozygotes. In principle, females heterozygous for *RpS3A ^57g^* can be used to establish disomic stocks, but the crosses would be less convenient, and the relatively low fecundity of these females would likely make the task more difficult.

The idea of distinguishing disomic flies from trisomic flies using a *Minute* mutation is not new. For example, Hawley et al. (1992) used a *Minute* mutation to screen for trisomic flies (though the authors did not report the identity of the specific *Minute* aberration or point mutation they used). After testing the use of *RpS3A ^57g^* for distinguishing disomic and trisomic flies, I found that Bushey and Locke (2004) had probably used it to assure flies in some of their crosses were disomic, but they did not elaborate on the idea. Whether or not I reinvented the wheel, I hope this approach proves valuable to anyone undertaking crosses involving the fourth chromosome.

## Methods

Fly cultures were reared under standard conditions. Genetic nomenclature reflects FlyBase release 2025_1 (Öztürk-Çolak et al. 2024). Information about the stocks listed in the Reagents section may be obtained via the websites for the Bloomington Drosophila Stock Center at Indiana University (https://bdsc.indiana.edu/) and the Department of Drosophila Genomics and Genetic Resources at the Kyoto Institute of Technology (https://www.dgrc.kit.ac.jp/).

## Reagents

**Table:** 

Stock number	Genotype	Available from	RRID
647	*ey ^1^*	Bloomington	RRID:BDSC_647
1229	*w ^1118^ ; RpS3A ^57g^ /In(4)ci ^D^ , ci ^D^ pan ^ciD^*	Bloomington	RRID:BDSC_1229
9422	*w ^1118^ ; Df(4)ED6369, P{3'.RS5+3.3'}ED6369/l(4)102EFf1/l(4)102EFf ^1^*	Bloomington	RRID:BDSC_9422
9549	*w ^1118^ ; P{w ^+mC^ =ActGFP}unc-13 ^GJ^ /In(4)ci ^D^ , ci ^D^ pan ^ciD^*	Bloomington	RRID:BDSC_9549
90851	y ^1^ w ^*^ ; TI{TI}Crk ^dsRed^ / In(4)ci ^D^ , ci ^D^ pan ^ciD^	Bloomington	RRID:BDSC_90851
90852	y ^1^ w ^*^ ; TI{GMR-HMS04515}Gat ^eya^ /In(4)ci ^D^ , ci ^D^ pan ^ciD^	Bloomington	RRID:BDSC_90852
150529	*w ^1118^ ; Df(4)ED6380, P{w ^+mW.Scer\FRT.hs3^ =3'.RS5+3.3'}ED6382/l(4)102EFf ^1/^ l(4)102EFf ^1^*	Kyoto	RRID:DGGR_150529
150531	*w ^1118^ ; Df(4)ED6382, P{w ^+mW.Scer\FRT.hs3^ =3'.RS5+3.3'}ED6382/P{w ^+mC^ =ActGFP}unc-13 ^GJ^ /P{w ^+mC^ =ActGFP}unc-13 ^GJ^*	Kyoto	RRID:DGGR_150531
150532	*w ^1118^ ; Df(4)ED6384, P{w ^+mW.Scer\FRT.hs3^ =3'.RS5+3.3'}ED6384/l(4)102EFf ^1^ /l(4)102EFf ^1^*	Kyoto	RRID:DGGR_150532
602664	w ^*^ ; Df(4)ED6380, P{w ^+mW.Scer\FRT.hs3^ =3'.RS5+3.3'}ED6380/In(4)ci ^D^ , ci ^D^ pan ^ciD^	Bloomington	RRID:BDSC_602664

## References

[R1] Baker Bruce S, Carpenter Adelaide T C (1972). GENETIC ANALYSIS OF SEX CHROMOSOMAL MEIOTIC MUTANTS IN
*DROSOPHILA MELANOGASTER*. Genetics.

[R2] Bushey Daniel, Locke John (2004). Mutations in Su(var)205 and Su(var)3-7 Suppress P-Element-Dependent Silencing in Drosophila melanogaster. Genetics.

[R3] Hawley R. Scott, Irick Holly, Haddox Deana A., Whitley Michelle D., Arbel Tamar, Jang Janet, McKim Kim, Zitron Anne E., New Christine, Childs Geoffrey, Lohe Allan (1992). There are two mechanisms of achiasmate segregation in
*Drosophila*
females, one of which requires heterochromatic homology. Developmental Genetics.

[R4] Hochman B., Gloor H., Green M. M. (1964). Analysis of chromosome 4 inDrosophila melanogaster. I. Spontaneous and X-ray-induced lethals. Genetica.

[R5] Kronhamn Jesper, Rasmuson-Lestander Åsa (1999). Genetic organization of the
*ci-M-pan*
region on chromosome IV in
*Drosophila melanogaster*. Genome.

[R6] Marygold Steven J, Roote John, Reuter Gunter, Lambertsson Andrew, Ashburner Michael, Millburn Gillian H, Harrison Paul M, Yu Zhan, Kenmochi Naoya, Kaufman Thomas C, Leevers Sally J, Cook Kevin R (2007). The ribosomal protein genes and Minute loci of Drosophila melanogaster. Genome Biology.

[R7] Morgan TH, Bridges CB, Sturtevant AH. 1925. The genetics of Drosophila. Gravenhage: M. Nijhoff.

[R8] Morgan TH, Sturtevant AH, Bridges CB, Schultz J. 1926. The constitution of the germ material in relation to heredity. Yearb Carnegie Instn Washington. 25:308-312.

[R9] Nyberg Kevin G., Nguyen Joseph Q., Kwon Yong-Jae, Blythe Shelby, Beitel Greg J., Carthew Richard (2020). A pipeline for precise and efficient genome editing by sgRNA-Cas9 RNPs in
*Drosophila*. Fly.

[R10] Öztürk-Çolak Arzu, Marygold Steven J, Antonazzo Giulia, Attrill Helen, Goutte-Gattat Damien, Jenkins Victoria K, Matthews Beverley B, Millburn Gillian, dos Santos Gilberto, Tabone Christopher J, Perrimon Norbert, Gelbart Susan Russo, Broll Kris, Crosby Madeline, dos Santos Gilberto, Falls Kathleen, Gramates L Sian, Jenkins Victoria K, Longden Ian, Matthews Beverley B, Seme Jolene, Tabone Christopher J, Zhou Pinglei, Zytkovicz Mark, Brown Nick, Antonazzo Giulia, Attrill Helen, Goutte-Gattat Damien, Larkin Aoife, Marygold Steven, McLachlan Alex, Millburn Gillian, Pilgrim Clare, Öztürk-Çolak Arzu, Kaufman Thomas, Calvi Brian, Campbell Seth, Goodman Josh, Strelets Victor, Thurmond Jim, Cripps Richard, Lovato TyAnna, FlyBase Consortium (2024). FlyBase: updates to the
*Drosophila*
genes and genomes database. GENETICS.

[R11] Ryder Edward, Ashburner Michael, Bautista-Llacer Rosa, Drummond Jenny, Webster Jane, Johnson Glynnis, Morley Terri, Chan Yuk Sang, Blows Fiona, Coulson Darin, Reuter Gunter, Baisch Heiko, Apelt Christian, Kauk Andreas, Rudolph Thomas, Kube Maria, Klimm Melanie, Nickel Claudia, Szidonya Janos, Maróy Peter, Pal Margit, Rasmuson-Lestander Åsa, Ekström Karin, Stocker Hugo, Hugentobler Christoph, Hafen Ernst, Gubb David, Pflugfelder Gert, Dorner Christian, Mechler Bernard, Schenkel Heide, Marhold Joachim, Serras Florenci, Corominas Montserrat, Punset Adrià, Roote John, Russell Steven (2007). The DrosDel Deletion Collection: A Drosophila Genomewide Chromosomal Deficiency Resource. Genetics.

[R12] Sturtevant A H (1936). PREFERENTIAL SEGREGATION IN TRIPLO-IV FEMALES OF DROSOPHILA MELANOGASTER. Genetics.

[R13] van Beest M (1998). Drosophila RpS3a, a novel Minute gene situated between the segment polarity genescubitus interruptus and dTCF. Nucleic Acids Research.

[R14] Weasner Bonnie M, Weasner Brandon P, Cook Kevin R, Stinchfield Michael J, Kondo Shu, Saito Kuniaki, Kumar Justin P, Newfeld Stuart J (2025). A new
*Drosophila melanogaster*
research resource: CRISPR-induced mutations for clonal analysis of fourth chromosome genes. G3: Genes, Genomes, Genetics.

